# Dyadic Neural Synchronization: Differences between Offline and Computer‐assisted Online Verbal Interaction

**DOI:** 10.1002/hbm.70436

**Published:** 2025-12-15

**Authors:** Shiang Hu, Piqiang Zhang, Yuhao Fang, Xiao Gong, Lihao Fu, Enguang Ma, Debin Zhou, Zhao Lv, Pedro A. Valdes‐Sosa

**Affiliations:** ^1^ Key Laboratory of Intelligent Computing and Signal Processing of Ministry of Education, Anhui Provincial Key Laboratory of Multimodal Cognitive Computation, School of Computer Science and Technology Anhui University Hefei China; ^2^ Stony Brook Institute at Anhui University Hefei China; ^3^ MOE Key Lab for Neuroinformation, School of Life Science and Technology University of Electronic Science and Technology of China Chengdu China; ^4^ Cuban Neuroscience Center Havana Cuba

**Keywords:** brain network, EEG hyperscanning, interpersonal neural synchronization, technological online communication, verbal interaction

## Abstract

Computer‐assisted online interaction (CAOI) has become predominant in daily life and is increasingly supplanting offline verbal interaction (FVI). Previous research has shown that face‐to‐face verbal interaction (VI) exhibits significant differences in interpersonal neural synchronization (INS) compared to computer‐assisted online VI. However, the differences between various forms of FVI and CAOI remain unclear. In this work, we designed different forms of naturalistic VI tasks between dual persons and adopted electroencephalography (EEG) hyperscanning to simultaneously record neural activities from both participants. The experiment included three conditions: online versus offline, with versus without feedback, with versus without visual information or eye contact. Thirty‐one pairs of labmates with ordinary levels of intimacy were recruited as subjects. To analyze the impacts of these VI forms on INS, we calculated intersubject correlation at both scalp and cortex levels and constructed brain‐to‐brain networks based on intersubject functional connectivity using the phase lag index at the scalp level and the phase locking value at the cortex level. We found that interactions with feedback exhibit higher synchronization than interactions without feedback. VIs with visual information or eye contact are more effective in eliciting stronger INS. Additionally, compared to FVI, CAOI exhibits weakened neural synchronization. Intriguingly, online text‐based interaction also results in high neural coupling. Our study reveals significant differences in various CAOIs and FVIs concerning typical factors, providing crucial insights into the mechanisms of INS during online interactions.

## Graphical abstract

This study systematically investigates neural synchronization in various forms of offline and online verbal interactions. It demonstrates that feedback and visual information similarly affect neural synchronization in online settings and reveals varying degrees of weakened neural synchronization in different online interactions compared to offline counterparts.

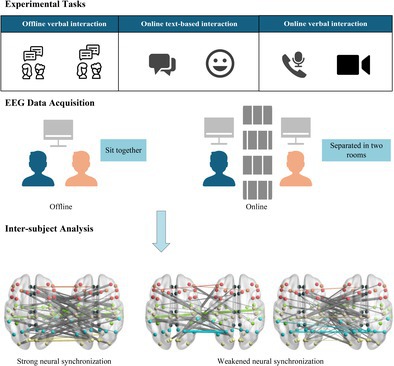

## 1 Introduction

An intention of social neuroscience is to understand the neural foundations behind social interactions, which requires studying naturalistic communication between people (Di Paolo and De Jaegher 2012; Hari and Kujala 2009). In social interactions, each individual's brain drives an action that elicits neural responses in their partner, leading to temporally contingent between‐brain coupling shaped by distinct interaction dynamics (Špiláková et al. 2020). Language communication plays a central role and has long been the focus of research in social interactions. Hyperscanning allows for the simultaneous recording of neural activities and information exchanges between multiple brains. Electroencephalography (EEG) is a preferred modality due to its affordability, noninvasiveness, mobility, and higher temporal resolution compared to other modalities dependent on blood oxygenation (Dikker et al. 2017; Kelsen et al. 2022; Michel and Brunet 2019; Nam et al. 2020), being capable of detecting brain development disorders and functional diseases (Li et al. 2022), as well as capturing changes in rapid neural processing during real‐world social interactions (Czeszumski et al. 2020). The core of our social experience is construed through real‐time interactions requiring the active negotiation of information with other people (García and Ibáñez 2014). The emergence of computer‐assisted online communication has transformed traditional face‐to‐face (F2F) conversations into remote ones, revolutionizing the form of verbal interaction (VI). Human VIs can range from ordinary dialogues to monologues such as in teaching (Dikker et al. 2017) and persuasion contexts (Zhang et al. 2023), and present various forms under the catalyst of digital communication techniques. Given the ubiquity and complexity of VI, studying their neural mechanisms using EEG hyperscanning is intriguing and meaningful.

Interpersonal neural synchronization (INS) refers to the temporal neural coupling between two or more individuals when they engage in a shared activity or social interaction (Finn et al. 2018). This coupling reflects the dynamic alignment of neural processes and is commonly measured through hyperscanning techniques such as EEG, fNIRS, or fMRI (Djalovski et al. 2021; Zhao et al. 2024). Recent research has revealed INS during VI, particularly during offline settings. Previous research on offline verbal interaction (FVI) has shown that brain oscillations are synchronized between the listener and the speaker during oral narratives (Pérez et al. 2017). Jiang et al. (2012) found a significant increase in INS in F2F dialogue but not in other types of offline interactions. From different perspectives of research interest, studies consistently demonstrate significant brain‐to‐brain coupling in the frontal, temporal, and parietal lobes during VI (Kelsen et al. 2022). Additionally, neural synchronization in VI is associated with multiple factors. First, INS is related to feedback or turn‐taking behavior. For example, an EEG hyperscanning study found significantly higher INS during the verbal turning number counting task compared to noninteractive tasks, highlighting the specific brain regions associated with turn‐taking behavior (Ahn et al. 2018). It also revealed that strong INS in the left frontotemporal and right centro‐parietal regions during VI may be associated with interactions involving feedback. Another study identified significant INS in language areas such as the superior temporal gyrus and the superior central sulcus during the interactive object naming task, suggesting that interaction with feedback modulates neural activity in these regions (Hirsch et al. 2018). Second, visual contact may play an important role during dyadic interaction. Hirsch et al. (2017) found stronger neural responses during eye contact compared to mutual gaze on a face picture, suggesting unique neural mechanisms activated by direct social interaction. Similarly, Kinreich et al. (2017) revealed that INS in the temporal–parietal regions was influenced by the level of intimacy and social gaze.

Since the COVID‐19 pandemic, computer‐assisted online interaction (CAOI) has exploded in popularity, manifesting in online meetings, courses, and remote work. This shift from traditional F2F interactions to digital platforms has transformed how we engage, influencing daily communication and raising questions about the quality of social interactions in a digital age. Virtual interfaces now permeate nearly every aspect of our lives. Balconi et al. (2022) found that the transition to CAOI has permeated all aspects of human social life after investigating the differences between F2F and CAOI. Their further research explored how remote, as opposed to F2F, training affects cognitive (such as memory and attention), affective, and social processes (Balconi et al. 2023). Schwartz et al. (2022) suggested that CAOI weakens mother–child INS compared to F2F interactions. Beyond remote VI, text messaging has emerged as a dominant mode of social interaction. Schwartz et al. (2024) indicated that the F2F interaction remains superior to online text for interpersonal connection.

Despite advances in means of VI, the differences in INS between FVI and CAOI remain unclear. Additionally, there is a lack of comprehensive research on various forms of VI. To address this, we used EEG hyperscanning to explore INS during different forms of VI, including online and offline conditions. Conducting hyperscanning studies based on EEG has traditionally relied on continuous interaction paradigms. However, ecological paradigms are more representative of real‐life interactions, making them more meaningful for studying social interactions (Acquadro et al. 2016). Based on this premise, we anticipated that online versus offline interactions, feedback versus no feedback, visual contact versus no visual contact would exhibit significant differences in INS. We expected higher INS in FVI compared to CAOI and stronger INS in VI with feedback or visual contact. Additionally, as a form of nonverbal online communication, online text‐based interactions (OTIs) have become a primary mode of social interaction, but their impact on social brain functioning remains to be further elucidated, and it might differ from verbal ones due to its inherent nature. We also incorporated OTI forms into our study, including plain text and text with emojis (TWEs). OTIs do not involve verbal conversation and are likely to involve distinct neural mechanisms. This form of virtual communication may reveal more about ourselves: for instance, individuals with higher emotional intelligence tend to use more emojis when communicating with friends, whereas those with an avoidant attachment style may use fewer emojis when interacting with others (Dubé et al. 2024).

This paper's key innovations stem from its comprehensive approach to ecologically valid experimental design with naturalistic stimuli and performing a systematic comparison of interaction forms, including interactions in online or offline settings, interactions with or without feedback, interactions with or without visual cues, and interactions through textual or verbal communication. This diverse design allows for a nuanced understanding of how different interaction modalities influence INS. Additionally, we utilized source analysis to further explore brain‐to‐brain synchronization, extending scalp analysis to cortex functional networks. This approach enabled us to compute INS from both scalp and cortex dimensions, providing a more detailed and multidimensional perspective on the neural mechanisms underlying interpersonal interactions. Ultimately, we broaden the research scope from examining the patterns of various forms of FVI to CAOI and conduct a more detailed analysis of the distinctions between online and offline interactions. This paper begins by detailing materials and methods, encompassing participant demographics, experimental paradigms, data acquisition, and preprocessing steps, alongside the methodologies for analyzing INS. Following this, the results section presents comprehensive findings from both scalp and cortical analyses. Then, we integrate these results in the discussion section, addressing any unexpected outcomes and providing a nuanced interpretation of the data. Additionally, we critically evaluate the study's limitations and offer insights into future research directions.

## 2 Materials and Methods

### 2.1 Participants

A total of 62 right‐handed subjects participated in the experiment, comprising 18 male–male pairs and 13 female–female pairs, with an age range of 19–27 years (mean age: 23.10 ± 1.43). Each pair of subjects knew each other and became acquainted for more than 6 months prior to the experiment. Both individuals in each pair had no mental disorders or psychological problems and had normal or corrected vision. Written informed consent was obtained from all subjects, and they were compensated with 80 RMB at the end of the experiment. The recruitment of human subjects and the following experimental protocols were approved by the Biomedical Ethics Committee of Anhui University.

### 2.2 Task and Procedure

**FIGURE 1:**
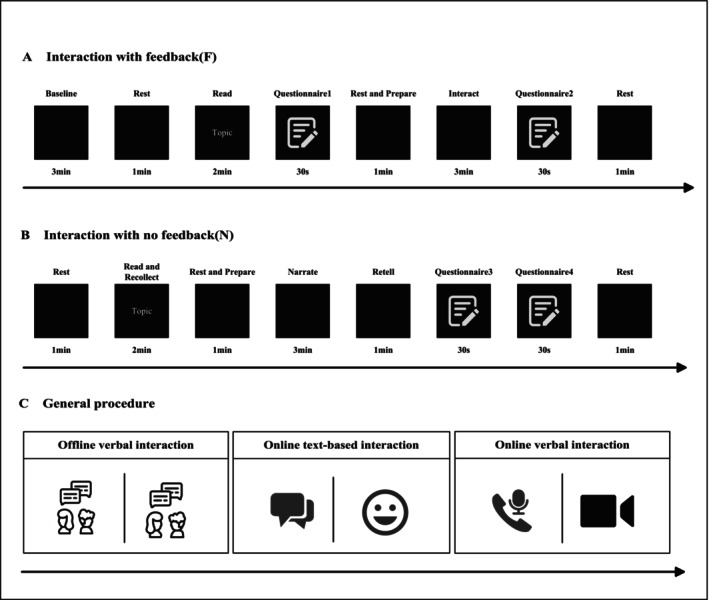
Experiment procedure. (A) Interaction with feedback. In the “interact” session, the brainwave activity of two participants was recorded for 3 min. (B) Interaction with no feedback. In the “narrate” session, the brainwave activity of two participants was recorded for 3 min. (C) General procedure. The experiment includes three sessions: FVI, OTI, and OVI.

This experiment adopted a multi‐factor design within the subject to investigate how different forms of intersubject natural interaction affect INS. The experimental paradigm utilized naturalistic stimuli, which closely mimic real‐world interactions, thereby enhancing the ecological validity of the study. This approach allows the research to more accurately reflect social interaction scenarios in the real world. As shown in Figure 1C, the forms of interaction generally fall into two categories: FVI and CAOI. In addition, FVI follows the form of a human face to human face (F2F) or the form of a human back to human back (B2B), the latter blocking facial expressions, eye contact, and other visual interactive factors. In contrast, a more elaborate setting is arranged for CAOI, with online verbal interaction (OVI) and OTI. The OVIs of the CAOI encompass voice calls (VoC) and video calls (ViC), while OTI contains two forms of plain text‐only (TO) and TWE. Both ViC and TWE that are set in CAOI are to mimic the F2F conditions in offline interactions by which the facial expressions are used.

Each pair of subjects first completed the basic information and familiarity assessment questionnaires upon entering the laboratory. Before EEG recording, all participants received instructions regarding the experiment guidelines. The topics were emotionally neutral, such as the current weather, the university events, and the use of AI tools. The corresponding keywords were displayed on the screen to ensure consistent understanding between pairs. Participants were asked to communicate objectively and factually and to avoid personal or emotionally charged content. All participants were also reminded to remain attentive but composed, avoiding unnecessary body or facial movements and exaggerated expressions, and to maintain a stable, calm posture throughout the tasks. At the initial resting‐state stage, the two subjects sat facing the wall without any interaction to record the resting EEG data for 3 min. During this period, no external emotional stimulus was applied, and the two subjects were simply instructed to relax for resting EEG recording. Thus, the emotional state of the baseline was considered neutral.

#### 2.2.1 Task 1: FVI

During FVIs, each of the F2F and B2B conditions was further divided into interactions with feedback and interactions without feedback.

During the interactions with feedback as shown in Figure 1A, the two subjects read a piece of news displayed on the screen in 2 min, then evaluated their familiarity with the topic using a 5‐point Likert scale in questionnaire 1 with 1 being the lowest level and 5 the highest. After scoring, there was a 1‐min break before participants were asked for a 3‐min discussion. To ensure ecological validity, the two subjects freely discussed previously read topics. The 5‐point Likert scale questionnaire 2 was used to evaluate their interactive quality. During interactions without feedback, as shown in Figure 1B, one of the two subjects was designated as the speaker, while the other was designated as the listener. The speaker was asked to self‐recollect personal experiences based on the topic prompt in 2 min. After a 1‐min break, the speaker narrated their experiences for 3 min, during which the listener kept listening only. After the narration, the listener was asked to repeat the speaker's statement and give brief comments. The speaker then rated the listener's level of understanding and the detail of their retelling using the 5‐point Likert scale questionnaires 3 and 4, respectively, and the listener themselves assessed their own level of listening and empathy as well. The interactions, regardless of feedback, were conducted in the forms of F2F and B2B. The two subjects were instructed to maintain eye contact in the F2F condition while looking at the wall in the B2B condition.

#### 2.2.2 Task 2: CAOI

CAOI includes four forms such as TO, TWE, VoC, and ViC, each of which was further set with and without feedback (response).

CAOI procedures were similar to the FVI except that the two subjects in CAOI were separated in two adjacent and soundproof rooms to mimic the remote scenario. In the TO condition, participants communicated by sending instant plain text messages through the WeChat app without using images, emojis, or voice. The length of the text was required to be at least a full sentence so that each message conveyed a complete meaning. In the TO condition with no feedback, the text sender sent a few consecutive sentences so that the reader could understand the content without replying. In the TO condition with feedback, the two subjects chatted in a turn‐taking way using full sentences and were asked to avoid unnecessary single‐word replies such as yes or no. In the TWE condition, participants followed the same requirement to send text messages in full sentences but ending with emojis, which were limited to the official default emoji package in the WeChat app. Emojis from third‐party packages were discouraged to control the type of emojis. In terms of emoji frequency, it was recommended to attach a few emojis at the end of the text in each message, but to avoid sending messages that contained only emojis or long emoji sequences as an “emoji battle.” In the VoC and ViC conditions, the two subjects communicated remotely by making voice or video calls through WeChat.

To alleviate the impacts of sequential orders across multiple conditions, we intended to set equal trials between with and without feedback, equal chances for a subject to be the speaker or listener, and randomize topic sequences. To control for content effects, we ensured that each interaction condition used the same set of topics. In addition, we balanced FVI and CAOI and alternated F2F and B2B conditions, which together helped minimize potential biases and enhanced the robustness of the experimental design.

### 2.3 Equipment and Data Acquisition

Two EEG amplifiers were used to simultaneously record EEG for a pair of subjects during the experiment. The same trigger was connected in parallel to the two EEG amplifiers to set the synchronous event marks. The EEG data were recorded in continuous wave form with a sampling rate of 500 Hz. For the first 16 out of 31 pairs of subjects, the two EEG amplifiers were the 32 channels mBrainTrain SMARTING Pro portable EEG systems, with online reference FCz (Hu, Karahan, et al. 2018). The EEG cap used the saline semi‐dry electrodes. Due to a malfunction in one of the two SMARTING Pro EEG systems, the remaining 15 pairs of subjects were recorded by the two Neuracle NeuSen‐W portable EEG systems consisting of 32 gel electrodes, whose electrode layout was consistent with the SMARTING Pro 32 system. Before starting EEG recording, the electrode impedance was reduced to less than 10 kΩ. Existing methods to harmonize the differences of EEG amplifiers are (1) the global scale factor removal (Hu, Ngulugulu, et al. 2019; Li et al. 2022); (2) applying the *z*‐score; (3) calculating the relative power (Hu, Xiang, et al. 2025), all of which are by means of normalization. The INS metric, either intersubject correlation (ISC) or intersubject functional connectivity (ISFC), inherently falls into the interval [0, 1] after normalizing. The first 15 of the total 30 pairs of subjects were recorded using the two identical SMARTING Pro EEG system, and the rest were recorded using the two identical NeuSen‐W EEG system. No mix of the two different systems was applied. Since subsequent analysis was mainly based on the normalized INS metrics from simultaneous dyadic EEG recordings, the influence of equipment differences between the first 15 pair of subjects and the rest 15 pairs of subjects becomes negligible.

### 2.4 EEG Preprocessing

Preprocessing significantly influences the results of post‐processing (Hu, Ruan, et al. 2025). The raw EEG data were preprocessed using EEGLAB, Brainstorm toolboxes, and custom MATLAB scripts. The preprocessing included the following steps: (1) down‐sampling to 250 Hz; (2) removing 50 Hz line noise by notch filtering; (3) average reference (Hu, Lai, et al. 2018; Hu, Yao, et al. 2019; Yao et al. 2019); (4) jaw movements during VI and muscle artifacts (Ahn et al. 2018) in mobile use were removed by independent component analysis (ICA); (5) extracting the trials of different experimental conditions according to their event marks; (6) bandpass filtering on the EEG signals resulted in the delta band (1–3 Hz), theta band (4–7 Hz), alpha band (8–12 Hz), and beta band (13–30 Hz) (Hu et al. 2024). After preprocessing, one pair of subjects was excluded due to poor data quality, resulting in a final sample of 30 pairs of subjects with valid data.

### 2.5 INS Analysis

**FIGURE 2:**
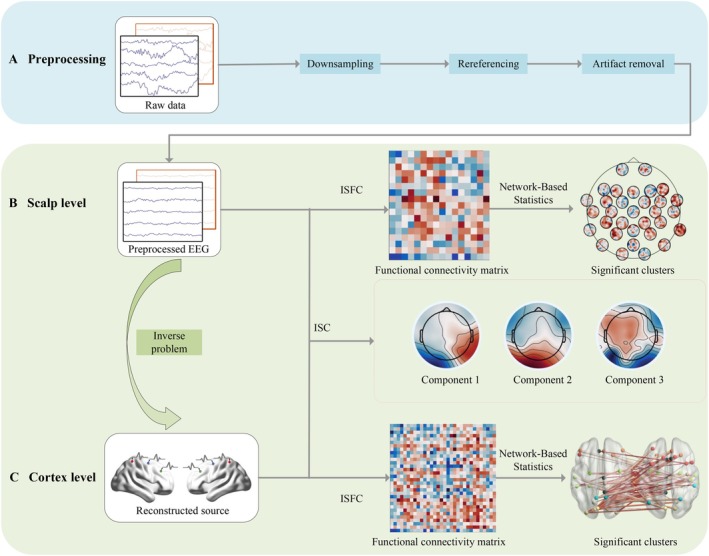
Workflow of dyadic EEG analysis. (A) Preprocessing. The raw data were first preprocessed, primarily involving downsampling, rereferencing, and artifact removal. (B) Scalp level. (C) Cortex level. The raw data, following a preprocessing step, were subjected to analysis in two dimensions: scalp‐level and cortex‐level. In each dimension, our analytical approach was similar. First, ISC was calculated at the individual level. Subsequently, functional connectivity matrices between electrodes or brain regions were constructed. Finally, a nonparametric clustering permutation test was employed to identify clusters of significant differences in brain networks across different conditions.

To measure INS, we utilized two approaches as shown in Figure 2: ISC at the entire brain level from temporal synchronization and ISFC from frequency synchronization at the network level. In addition, to assess ISFC between brain regions, source localization was performed to construct cortical functional networks.

#### 2.5.1 ISC

At the entire brain level, ISC was a classic measure for quantifying intersubject temporal synchronization. Performing the correlated component analysis (CCA) (Parra et al. 2019) on dyadic EEG from a pair of subjects can identify a linear combination of the neural activities over electrodes or sources and present the maximum correlation between the two subjects. Different from the principal component analysis (PCA), the CCA extracts the projection with the highest correlation rather than with the highest variance. ISC was calculated individually to determine the similarity within individual pairs of subjects in responses to the same stimulus. ISC represents the sum of the first three maximum correlations in all components, with higher values indicating greater similarity in responses between participants (Cohen and Parra 2016).

#### 2.5.2 ISFC

Previous studies have attempted to identify functional connectivity using EEG (Xu et al. 2024). A critical consideration in our choice of connectivity metrics is the volume conduction effect, a common challenge in EEG signal analysis. Volume conduction refers to the passive spread of electrical potentials through the brain, skull, and scalp, which can result in spurious zero‐lag correlations between signals. To mitigate this, we adopted the phase lag index (PLI) for scalp‐level ISFC, as it can minimize the influence of common sources—an instantaneous effect by ignoring phase synchrony at zero‐lag and only capturing the nonzero phase differences. This makes PLI a reliable indicator of genuine functional connectivity in scalp EEG recordings (Stam et al. 2007). In contrast, at the cortex level, where source reconstruction techniques significantly reduce the impact of volume conduction, we adopted the phase locking value (PLV). PLV captures the full strength of phase synchronization, including potentially meaningful zero‐lag interactions. It is particularly sensitive to consistent phase relationships across brain regions and has been widely used to characterize interregional coupling in source‐reconstructed EEG studies (Lachaux et al. 1999; Olejarczyk and Jernajczyk 2017). Although the intra‐subject network is also interesting and worthy of analysis, the focus of this study is on INS. Therefore, we report only the intersubject results.

#### 2.5.3 Scalp‐Level ISFC

PLI is a metric used to study phase coupling, and it provides information about the phase synchronization or phase differences between different recording sites. Phase coupling reflects the temporal consistency of phase relationships. We can calculate it using the following formula (Stam et al. 2007):

(1) PLI=n−1∑t=1nsignImeiϕj−ϕkt

where n is the number of timepoints, $\phi^{j}$ and $\phi^{k}$ represent the phase at sensor j and k, t represents the timepoint. So $\phi^{j}-\phi^{k}$ represents the phase differences between sensor j and sensor k. Imeiϕj−ϕkt is the imaginary part of the vector in the complex plane. And $\mathrm{sign}\left(x\right)$ is a signum function, taking the value of 1 when x is greater than 0 and −1 when x is less than or equal to 0.

The phase information per frequency of each electrode pair was extracted by applying a Hilbert transform. For each time point, the phase difference between the electrode pairs was computed. The sign of the phase difference was determined for all time points. This Formula (1) computes the average sign difference of the phase disparity between two signals, yielding a result between 0 and 1. A PLI value closer to 1 indicates a higher degree of phase synchronization between the two signals.

We constructed the ISFC for each condition and made comparisons between three sets of conditions using network‐based statistical (NBS) analysis in the scalp networks that were the interactions with versus without feedback, interactions with versus without visual information or eye contact, online versus offline interactions.

#### 2.5.4 Source Estimate and Cortex ISFC

In the source analysis, we selected interaction conditions that are representative of everyday communication and ensure consistency in visual information across FVI, OTI, and OVI. Therefore, we further investigated the interaction conditions F2F, TWE, and ViC, all with feedback. To further explore the underlying neural mechanisms, we applied the source localization technique, sLORETA (Pascual‐Marqui 2002), which is a linear distributed source solution. It can be represented as Y=AX, where Y represents the filtered EEG signals obtained by applying the Section 2.4, Step (6), X represents the source activities distributed in the cortical space, and A is the lead field gain matrix estimated by the forward solver with an appropriate head model. The electrode positions and labels were calibrated and corrected after importing EEG recordings into Brainstorm. The anatomical template ICBM152 was used to estimate the head model and the lead field gain matrix using the boundary element method with the OpenMEEG tool. Finally, the high‐dimensional source time series were mapped and assigned to 62 brain regions according to the Desikan–Killiany–Tourville atlas (Klein and Tourville 2012) after extracting the main component from source activities ascribed to each brain region using the dimensionality reduction technique PCA. The 62 brain regions are distributed throughout the prefrontal lobe, frontal lobe, parietal lobe, occipital lobe, central region, temporal lobe, and limbic cortex. We then calculated the PLV for the extracted source time series from each pair of brain regions to obtain cortex‐level interregional functional connectivity after source reconstruction, which served as the basis for the subsequent graph‐theoretic network analysis (Ismail and Karwowski 2020; Schmidt et al. 2014). First, the phase difference was quantified as the degree of phase synchronization between two regions of the brain, reflecting interregional functional connectivity. The traditional PLV was then calculated by averaging the phase differences across event‐related trials, reflecting the inter‐trial variance of phase differences. The time‐averaged PLV is a suitable variant adapted to EEG hyperscanning studies by calculating the average of instantaneous phase differences over time in a single trial (Burgess 2013) as follows:

(2) $${\mathrm{PLV}}_{t}=\frac{1}{T}\left|\sum_{n=1}^{T}e^{i\left(\phi_{\left(t,n\right)}-\psi_{\left(t,n\right)}\right)}\right|$$

where T is the number of time points, $\phi_{\left(t,n\right)}-\psi_{\left(t,n\right)}$ represents the instantaneous phase difference between different brain regions. The PLV ranges from 0 to 1, with 1 indicating perfect phase locking and 0 indicating no phase locking.

Under conditions F2F, TWE, and ViC, the time‐averaged PLV was applied to construct interbrain functional connectivity matrices. Subsequently, the thresholding effect was applied to remove trivial connections. In the cortex‐level analysis, we further examined functional connectivity and divided the brain into seven areas, comprising 62 regions of interest (ROIs) employing the Desikan–Killiany–Tourville atlas. Subsequently, we conducted comparisons between the three interaction conditions and the resting state. The ISFC patterns were then visualized using the BrainNet Viewer (Xia et al. 2013) to illustrate the significant differences in interbrain functional connectivity using NBS. In addition, we used the Brain Connectivity Toolbox to compute graph‐based network metrics (Rubinov and Sporns 2010), including average node degree, clustering coefficient, transitivity, global efficiency, and local efficiency, which enabled us to further explore the characteristics of brain networks and quantify patterns of interbrain connectivity.

### 2.6 NBS Analysis

Since this study is a hypothesis‐driven analysis and the multiple comparison problem needs to be solved, we use a nonparametric clustering permutation method to compare the INS differences across interactive conditions. A nonparametric cluster permutation test was employed for the NBS analysis (Maris and Oostenveld 2007; Zalesky et al. 2010). To mitigate the potential bias of the distribution assumptions in the parametric test, the distribution of topological differences of the brain networks under two interaction conditions was established through condition label randomization and network‐based clustering criteria, extracting the t‐statistic in a data‐driven manner. The large number of possible connections in brain networks easily causes the multiple comparison problem. The family‐wise error rate (FWER) is the probability of making at least one false positive error when conducting multiple statistical tests. The nonparametric cluster permutation test can address the FWER problem by considering the topological structure of the brain network, that is, by evaluating clusters of edges that show coherent patterns but not testing each isolated edge. Initially, we calculated the statistical difference for each edge and excluded absolute *t* values less than 1.96. The remaining edges were then clustered into strongly connected components (SCCs) based on whether they reflected similar effects (separate clusters for positive and negative edges). Subsequently, the difference distribution curves for the condition differences were estimated using 5000 permutations by randomly shuffling the condition labels while preserving the integrity of the data under two interaction conditions. In each iteration, we computed the sum of t‐scores within each cluster and retained the absolute maximum cluster score as the cluster t‐statistic. The *t*‐critical values were calculated to align with a two‐tailed significance level of 0.05. The clusters formed by the actual labels with t scores that exceeded the *t*‐critical values were ultimately identified by SCC‐wise inference on the difference distribution. This approach (Luft et al. 2022) was applied to all cluster analyses presented in this paper, both at the scalp and cortex levels.

## 3 Results

### 3.1 Statistical Assessment of the Experimental Paradigm

#### 3.1.1 Questionnaire Evaluation

The questionnaire 1 during the interactions with feedback was analyzed to assess the familiarity of subjects with six topics. There were no significant differences within either the six topics or between the two subjects (*p* = 0.264 > 0.05). After interactions with feedback, subjects were asked to assess their communication quality. There was a significant difference between F2F and B2B interactions (*p* = 0.028 < 0.05), but no significant differences were found between the two subjects (*p* = 0.637 > 0.05). The differences in ratings between different interaction conditions were statistically significant. These results indicate that F2F interactions with feedback represent a higher interaction quality compared to B2B interactions with feedback, and the two subjects in each pair had similar perceptions of their communication quality.

In the interactions without feedback, to ensure that the listeners were attentively listening to the speaker's monologue, the listeners were asked to immediately retell the main points of the speaker's monologue when the speaker finished narrating. All listeners were able to adequately repeat the main points.

#### 3.1.2 Sample Size Justification

Note that the “Feedback” factor was taken as the elementary factor to reproduce in the experiment design as it is widely agreed (Christoffels et al. 2007; Kulik and Kulik 1988), while the “Information_Type” and “Eye_Contact” were considered the primary factors to investigate in this study. Using G*Power v3.1, a sensitivity power analysis was conducted to compute the required effect size. Given our final sample size (*N* = 30), an *α* level of 0.05, and a power of 0.80, our study was sufficiently powered to detect a minimum detectable effect size of 0.529 (Faul et al. 2009). Furthermore, we calculated the observed effect size for the elementary factor “Feedback,” which was based on the mean values and standard deviations of the scalp‐level ISC values in the delta band. The observed effect size was 0.767 according to Cohen's $d_{\mathrm{rm}}$ (Lakens 2013). Given that our observed effect size (0.767) is larger than the minimum detectable effect size identified by the sensitivity analysis (0.529), this suggests our sample size was sufficient to reproduce the elementary “Feedback” factor. If taking the post hoc analysis given the actual effect size 0.767, *α* level 0.05, and sample size 30, we obtained the power 0.982, which is larger than the power threshold value 0.8. This together indicates that the sample size of 30 was well‐powered to find the between‐group difference.

#### 3.1.3 Residual Effects

To improve the reliability of the trial, we varied the interaction sequence for specific subject pairs. This adjustment helps counteract the residual effects of fatigue and habituation that a fixed sequence can produce. We tested for the presence of these residual effects using a linear mixed‐effects model (DeBruine and Barr 2021). We used scalp‐level ISC values as the response variable, the “Feedback” factors (WF, NF), the “Information_Type” factors (Meeting, Text, Call), the “Eye_Contact” factors (Y, N) of the current stimulus trial as the fixed effects, the three‐factor conditions in the previous stimulus trial as the random effects. Besides, the scaled “trial_number” was modeled as random effect as well to validate whether the residual effect is caused by time. The baseline model, which assumes that the current ISC is solely influenced by the conditions of the present trial, was specified as $\mathrm{ISC}∼\text{Feedback}\times \text{Information}\_\text{Type}\times \mathrm{Eye}\_\text{Contact}+\left(1|\text{subject}\right)$. To compare against the baseline model, we developed an extended model that incorporates carryover effects. It was specified as follows: *ISC*~*Feedback* * *Information_Type* * *Eye_Contact* + *Pre_Feedback*: *Feedback* + *Pre_InformationType*: *InformationType* + *Pre_EyeContact*: *EyeContact* + (1|*subject*), incorporating carryover effects for comparison with the baseline model.

The comparison between the two models yielded a highly nonsignificant result (*p* = 0.8635 > 0.05), indicating that no residual effects were present in the experiment.

### 3.2 Scalp and Cortex ISC Across Conditions

**FIGURE 3:**
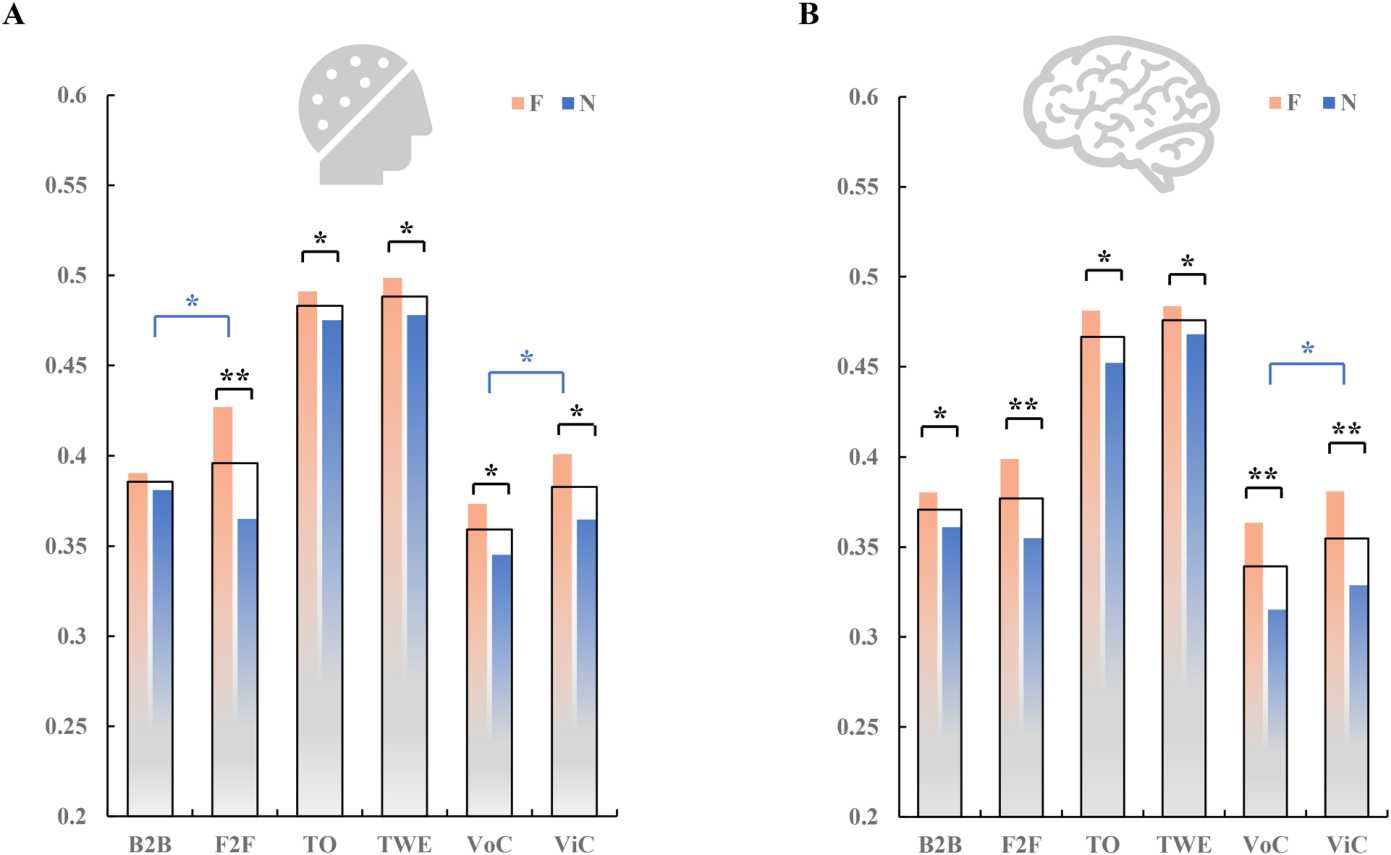
ISC comparisons across six interaction conditions in delta band. (A) Scalp‐level ISC. (B) Cortex‐level ISC. F: interactions with feedback; N: interactions without feedback. The black rectangles covering the F and N conditions indicate the mean ISC of the F and N conditions. Black asterisks indicate statistical significance between the F and N conditions. Blue asterisks indicate statistical significance between the conditions corresponding to the black rectangles. “*” indicates *p* < 0.05, and “**” indicate *p* < 0.01.

At both scalp and cortex levels, the ISCs were calculated to identify the differences in INS under each interaction condition. The FVI and CAOI included six interaction conditions that were F2F, B2B, TO, TWE, ViC, and VoC, each of which was examined with or without feedback. For each pair of subjects, scalp‐level ISC was computed for all six interaction conditions and then averaged to yield one mean ISC per frequency band. Only the delta band ISC showed significant differences (scalp: p = 0.0002 < 0.05) between the feedback and no‐feedback conditions, whereas the theta band (scalp: p = 0.080 > 0.05), alpha band (scalp: p = 0.121 > 0.05), and beta band (scalp: p = 0.478 > 0.05) did not show significant results. Prior hyperscanning studies have highlighted the importance of delta band for interbrain synchronization across a range of contexts (Müller and Lindenberger 2023; Sänger et al. 2012). Consequently, throughout this paper, all analyses are based on delta band. As shown in Figure 3A,B, interactions with feedback (F) in orange bars consistently exhibited higher ISCs compared to interactions without feedback (N) in blue bars under six interaction conditions. This indicates that interactions with feedback often lead to higher INS.

To investigate the impacts of visual involvement, such as facial expressions, eye contact, and emojis used in messages, we deliberately compared B2B and F2F in FVI, TO and TWE in computer‐assisted OTI, and VoC and ViC in computer‐assisted OVI. As shown in the comparisons between the dark rectangles of Figure 3A,B, interactions involving visual information showed higher mean ISC, whether with or without feedback, compared to interactions without visual information, both at the scalp and cortical levels. Except for no significant differences between TO and TWE, F2F showed a higher mean ISC than B2B (scalp: p = 0.038 < 0.05), and ViC showed a higher mean ISC than VoC (scalp: p = 0.021 < 0.05, cortex: p = 0.027 < 0.05). This indicates that the involvement of visual interactive information has a significant enhancing effect on increasing INS between the two brains.

Additionally, within the FVI, F2F F showed the highest ISC among its four internal conditions. In OTI, TWE F exhibited the highest ISC. In OVI, ViC F demonstrated the highest ISC. This suggests that both feedback and visual information during interactions play crucial roles in promoting higher INS. Among the six interaction conditions, TWE exhibited the highest mean ISC both in scalp and cortex levels, which can be seen from the highest black rectangle as shown in Figure 3A,B. Intriguingly, TO N and TWE N were both significantly higher than F2F F, indicating that visual interaction through textual information enhances dyadic neural synchronization more effectively than F2F visual interaction.

Comparing the results from the scalp and cortex levels as depicted in Figure 3B, the comparisons at the cortex yielded consistent results with those at the scalp level. It was observed that interactions with feedback (F) exhibited higher ISCs than those with no feedback(N). Regarding visual involvement, only ViC demonstrated a statistically significant difference compared to VoC; however, on average, F2F and TWE displayed stronger ISC values than B2B and TO, respectively. Regarding the comparison between CAOI and FVI, the findings were consistent with those presented in the scalp: OTI showed stronger ISC values than both FVI and OVI; furthermore, FVI exhibited higher ISCs compared to OVI. Collectively, the ISC analysis at the scalp and cortex levels suggested that: (1) OTI > FVI > OVI; (2) interaction with feedback was always better than without feedback; and (3) visual engagement during interaction showed a significant increase in OVI/FVI compared to OTI.

### 3.3 Differences in Scalp ISFC of Neural Synchronization Between Conditions

**FIGURE 4:**
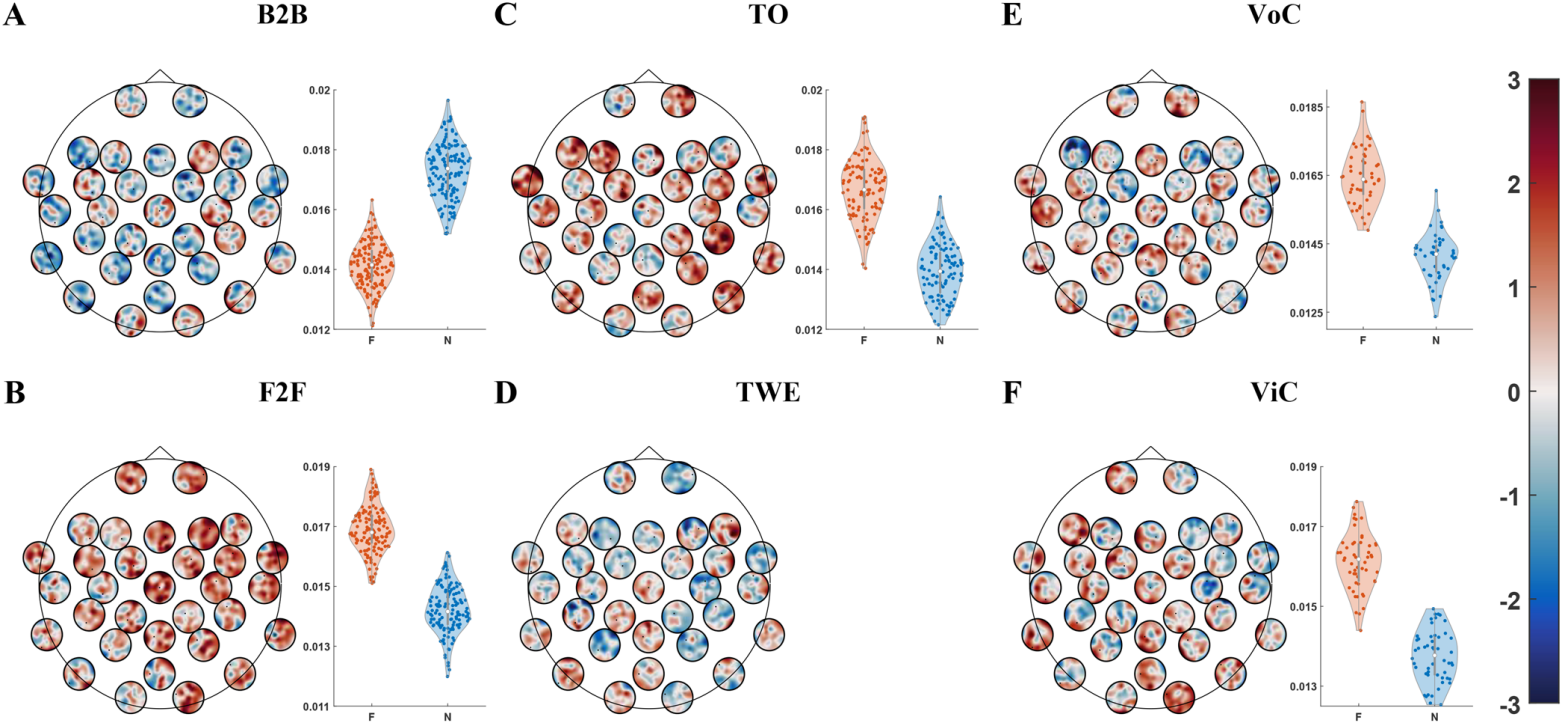
Comparison of ISFC between interactions with and without feedback. (A–F) Each subplot is divided into two parts and represents a comparison of interactions with and without feedback across six conditions (B2B, F2F, TO, TWE, VoC, ViC). The left part of the figure shows significant differences in functional connectivity between interactions with and without feedback. It employs a head‐in‐head plot design, wherein the large circle symbolizes the brain of one participant. Within this large circle, each smaller circle illustrates the functional connectivity topography between the electrode at that specific brain location and the 32 electrodes distributed across a different participant's brain. The colors inside the smaller circles denote intensity levels, which are referenced by the color bar on the far right. The values represent *t* statistics, where t > 1.96 indicates significantly greater functional connectivity in interactions with feedback compared to those without feedback, and t < −1.96 indicates the opposite. The right part of the figure displays violin plots showing the distribution of PLI values for electrode pairs that show significant differences between interactions with and without feedback. Since no significant connections were found between the F and N conditions in the TWE interaction, this section is left blank. All connections are statistically significant based on nonparametric cluster permutation tests.

The impact of feedback in online and offline interactions is also evaluated in brain network analysis, as shown in Figure 4. At the scalp level, we compared the effects of feedback participation on functional connectivity between brains under various conditions. After a network‐based nonparametric clustering test, our findings indicate that, apart from B2B and TWE conditions (Figure 4A,D), interactions with feedback (F) showed stronger and more extensive functional connectivity than interactions without feedback (N) under other conditions (Figure 4B,C,E,F). In the B2B condition, interactions without feedback (N) showed significant and stronger ISFC than interactions with feedback (F). No significant functional connectivity was found between F and N conditions in the TWE interaction. Overall, these findings emphasize the facilitating role of feedback in modulating INS.

**FIGURE 5:**
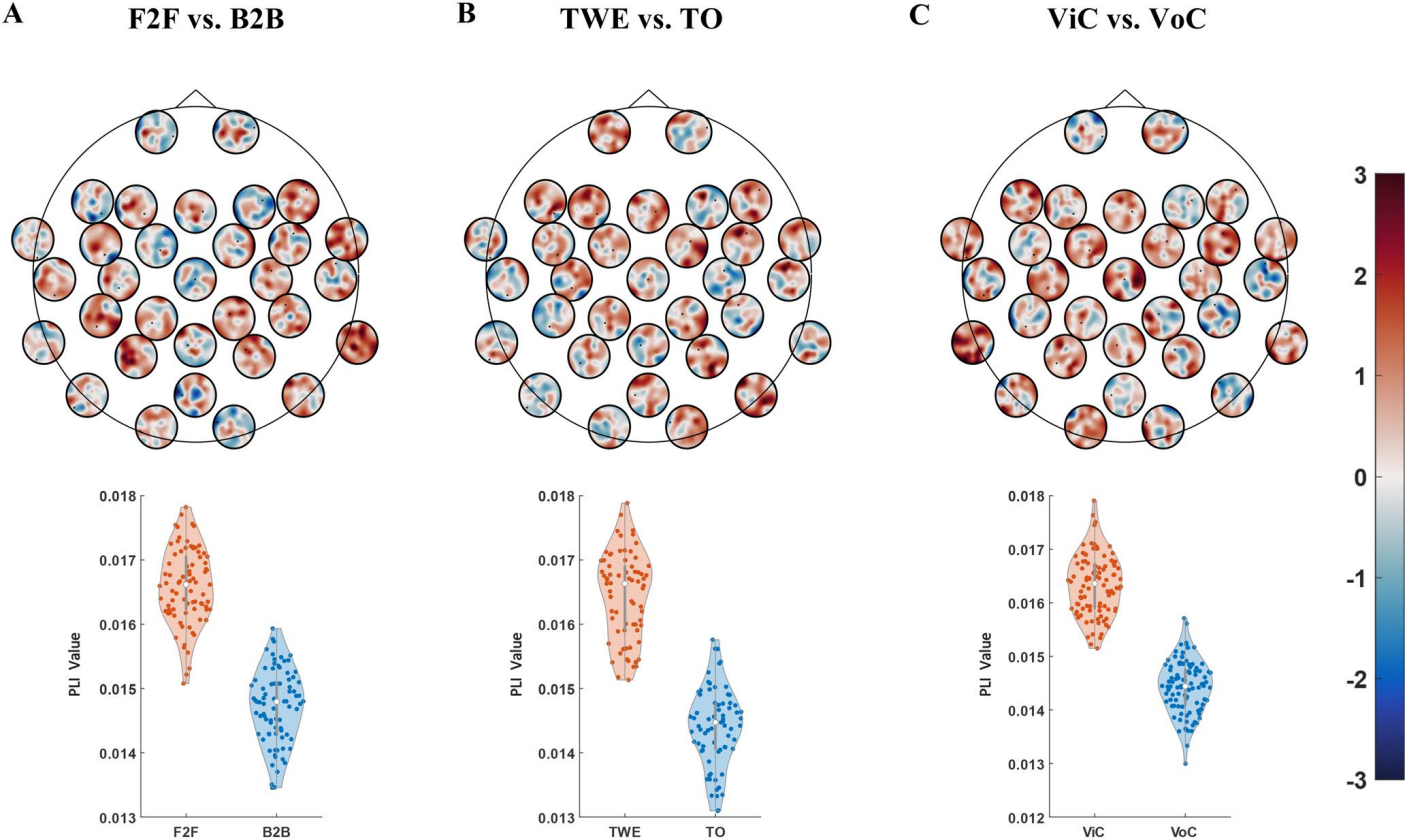
Comparison of ISFC between interactions with or without eye contact or visual information. (A–C) Each subplot is divided into two parts and represents a comparison of interactions with and without eye contact or visual information across three types of interaction: FVI (F2F vs. B2B), OTI (TWE vs. TO), and OVI (ViC vs. VoC). For all three conditions, the former scenario in each pair involves eye contact or visual information, while the latter does not. The upper part of each subplot shows significant differences in functional connectivity. The values represent *t* statistics, where t > 1.96 indicates significantly greater functional connectivity in the former (with eye contact or visual information) compared to the latter, and t < −1.96 indicates the opposite. The lower part of each subplot displays violin plots showing the distribution of PLI values for electrodes that exhibit significant differences between the two conditions. All connections are statistically significant based on nonparametric cluster permutation tests.

The impact of the participation of visual information in interactions is also evaluated in the analysis of brain networks, as shown in Figure 5. At the scalp level, we compared the effects of visual information participation on interbrain functional connectivity across three groups (F2F vs. B2B, TO vs. TWE, ViC vs. VoC). We found that interactions with visual information exhibited stronger and more extensive interbrain functional connectivity. It confirmed the importance of visual contact in FVI, and the phenomenon also holds in CAOI, indicating that meaningful social connections can still be formed even in virtual settings.

**FIGURE 6:**
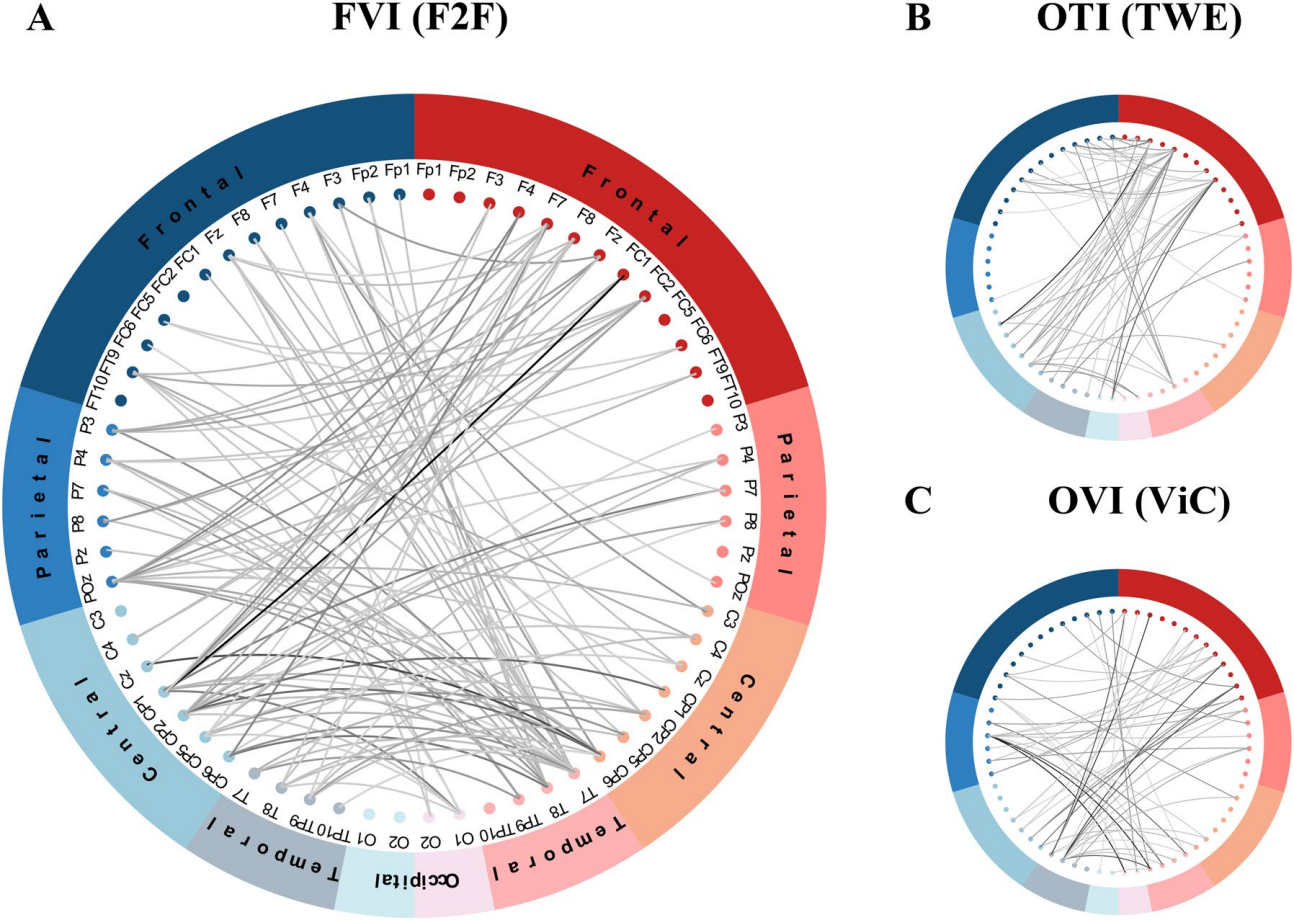
Comparison of online and offline interactions with the resting state. (A) FVI (F2F). (B) OTI (TWE). (C) OVI (ViC). (A–C) Each ring is divided into left and right halves, representing the scalps of two subjects. Each half of ring is divided into five sections which are the frontal lobe (F), parietal lobe (P), central region (C), temporal lobe (T), and occipital lobe (O). The nodes nested in the inner circle are the electrodes distributed into each brain region. The lines between dots indicate significant differences in functional connectivity compared to the resting state. The color of the lines ranges from gray to black, with darker colors indicating more significant differences. All connections are statistically significant based on nonparametric cluster permutation tests.

In the comparison between FVI and OVI as shown in Figure 6, we observed stronger interbrain functional connectivity during FVI (Figure 6A) compared to OVI (Figure 6C). Both FVI and OVI exhibited stronger interbrain functional connectivity than OTI (Figure 6B). These findings indicate that, compared to online interactions, offline interactions, with direct emotional cues, facial expressions, and eye contact, can significantly enhance INS. The physical presence in offline interactions may facilitate deeper emotional resonance and social connections, thereby promoting stronger functional connectivity.

### 3.4 Differences in Cortex ISFC of Neural Synchronization Between Conditions

**FIGURE 7:**
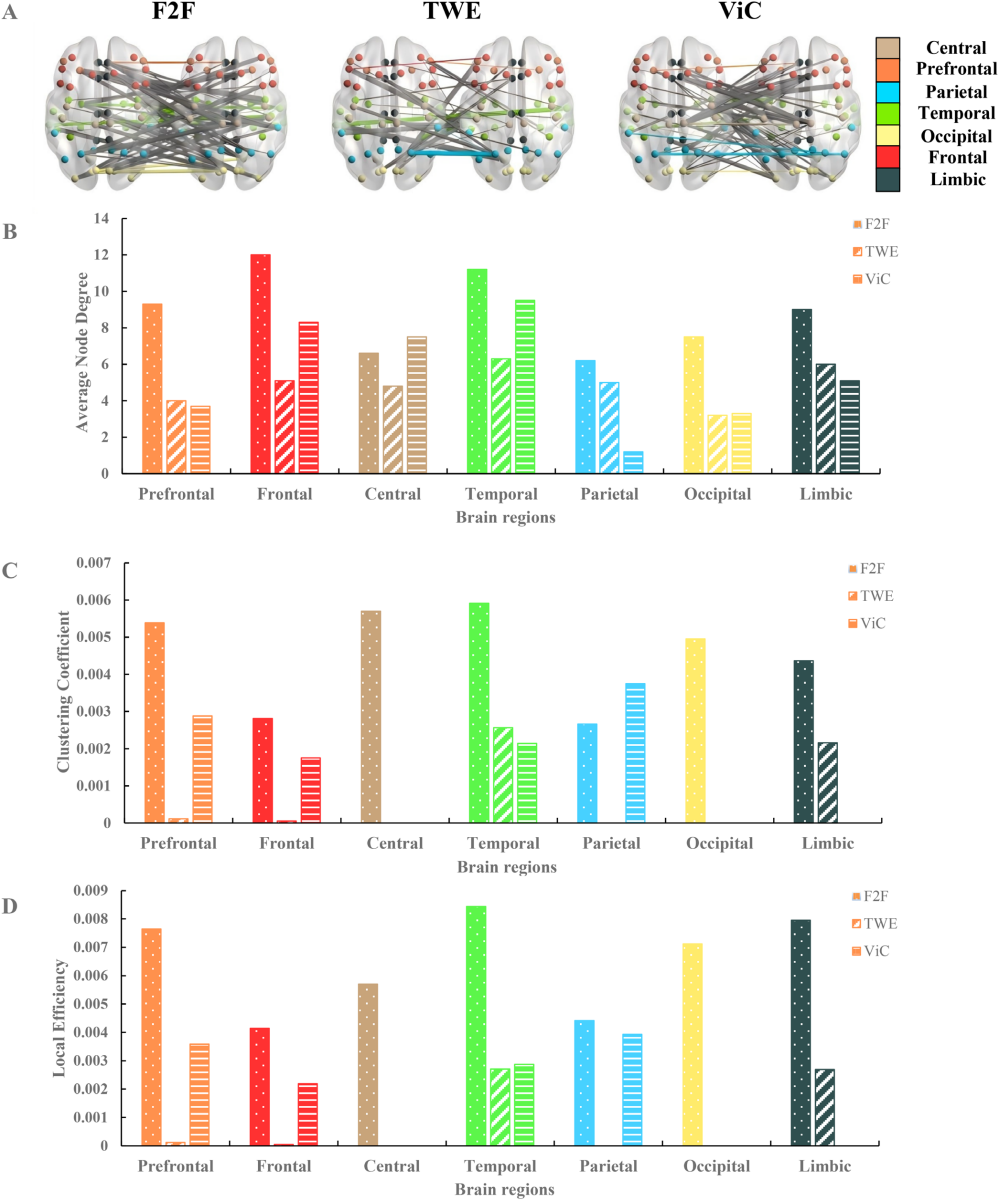
Comparison of F2F, TWE, and ViC interactions with resting state. (A) Significant positive interconnections of F2F, TWE, and ViC interactions agsinst the resting state. Different colored dots on the brains represent different brain regions, with the corresponding colors referenced in the color bar on the far right. The lines connecting the dots indicate connections that show significant differences compared to the resting state, meaning that the functional connectivity between these nodes is significantly higher under the given condition than during resting state. Colored lines represent connections between the same brain regions of two participants, while gray lines indicate connections between different brain regions. The thickness of the lines represents the strength of the functional connectivity. (B–D) Average node degree, clustering coefficient and local efficiency of different brain regions across three types of interaction. Seven colors represent different brain regions. The bars with white dots, white diagonal stripes, and white horizontal stripes represent F2F, TWE, and ViC interactions, respectively.

In the analysis of cortex ISFC, we compared FVI (F2F), OTI (TWE), and OVI (ViC) with the resting state, as shown in Figure 7A. We found that, compared to the resting state, both offline interaction (F2F) and online interactions (TWE and ViC) exhibited neural synchronization. When comparing the number of significant connections between the three interaction types, we found that the interbrain FC is densest for FVI, followed by OVI, and the interbrain FC is sparest for OTI. At the whole brain level, we calculated the clustering coefficient, transitivity, global efficiency, and local efficiency and found that each metric significantly differed across conditions (all *p* < 0.05). The clustering coefficient of F2F is highest among the three conditions, indicating the strongest local synchrony between individuals. Transitivity shows a similar trend, indicating a more cohesive neighborhood structure in F2F than in ViC and TWE. The global and local efficiency also show the same ordering, reflecting the information‐transfer capacity at global and neighborhood scales (Astolfi et al. 2020). At the nodal level, F2F showed significantly greater activation compared to TWE in several regions, including the superior frontal gyrus, entorhinal cortex, superior parietal gyrus, inferior parietal gyrus, lateral occipital cortex, supramarginal gyrus, caudal middle frontal gyrus, and insular cortex. Additionally, the superior frontal gyrus, middle temporal gyrus, and rostral anterior cingulate cortex were more active in F2F than in ViC. At the edge level, compared to TWE, F2F showed more widespread neural activation with interconnected edges between regions such as limbic–temporal, frontal–occipital, limbic–occipital, frontal–temporal, temporal–parietal, prefrontal–temporal, and limbic–limbic, indicating greater neural network connectivity across multiple regions in F2F. In contrast, compared to ViC, F2F also exhibited stronger connectivity between edges like limbic–parietal, frontal–occipital, frontal–limbic, temporal–prefrontal, temporal–parietal, and frontal–parietal, reflecting different connectivity patterns between F2F and ViC at the network level. Our observations indicated that brain regions associated with social cognition and emotional processing demonstrated particularly dense connectivity.

To conduct a more detailed investigation of the connections between brain regions, we calculated graph‐based metrics of interregional brain networks, and we found significant differences in the average node degree, clustering coefficient, and local efficiency (all *p* < 0.05). As shown in Figure 7B, we observed that offline interaction (F2F) exhibited a significantly higher number of functional connections across seven brain regions compared to online interactions (TWE and ViC), suggesting denser interbrain communication and broader spatial activation in the cortical space. For example, the frontal lobe, temporal lobe, and prefrontal cortex showed stronger activation during F2F interactions, underscoring the unique role of physical presence in enhancing neural synchronization. Additionally, within online interactions, we observed a higher number of significant connections in the frontal lobe, temporal lobe, and central region during ViC. In contrast, TWE exhibited more significant connections in the temporal lobe, parietal lobe, and limbic cortex. Figure 7C,D shows that both interregional clustering coefficient and local efficiency demonstrated consistent results with higher values for F2F than TWE and ViC, particularly in all regions, indicating denser clustering and more efficient information transfer between participants in the F2F condition. ViC shows relatively elevated values in the prefrontal, parietal, and temporal regions. However, TWE only markedly exhibits increases in the temporal and limbic regions.

## 4 Discussion

Our study investigated how various forms of VI influence INS, examining both offline and online settings with and without feedback, as well as with and without visual information or eye contact. To provide a concise overview of the findings, we first summarize the relative ranking of INS across interaction modalities (Table 1). Using EEG hyperscanning, we calculated ISC and ISFC at both scalp and cortical levels, leading to several key insights: (1) Turn‐taking, real‐time, bidirectional exchanges of information significantly enhance INS. Feedback interactions consistently exhibited higher INS compared to those without feedback, underscoring the importance of turn‐taking behavior in social interactions. (2) Visual contact is crucial for higher INS. F2F and ViC interactions induced significantly greater INS than B2B conditions and VoC, highlighting the role of mutual gaze and visual cues in social engagement. Although no significant differences were found between OTI with and without emojis, this may be attributed to the uncontrolled nature of emoji use in our ecologically valid experimental design. (3) CAOI tends to reduce the level of INS typically observed in natural social interactions. We observed that natural FVI promoted higher INS compared to CAOI.

**TABLE 1:** Summary of INS ranking across interaction modalities.

	Analysis dimension	INS ranking
ISC	Scalp and cortex‐level (Figure 3)	TWE_F > TO_F > TWE_N > TO_N > F2F_F > ViC_F > B2B_F > B2B_N > VoC_F > ViC_N > F2F_N > VoC_N
ISFC	Scalp‐level (Figures 4, 5, 6)	*Feedback (F) and Without feedback (N)* F > N (F2F, TO, VoC, ViC), N > F (B2B) *Visual contact* F2F > B2B, TWE > TO, ViC > VoC
	Cortex‐level (Figure 7A,B)	F2F Frontal > Temporal > Prefrontal > limbic > Occipital > Central > Parietal *TWE* Temporal > Limbic > Frontal > Parietal > Central > Prefrontal > Occipital *ViC* Temporal > Frontal > Central > Limbic > Prefrontal > Occipital > Parietal *Degree comparison within brain region* F2F > TWE > ViC (prefrontal, occipital, parietal, limbic), F2F > ViC > TWE (temporal, frontal), ViC > F2F > TWE (central)
Multifactor	Feedback Visual contact Information type	Feedback > Without feedback Visual > Without visual FVI > OVI > OTI

### 4.1 Interaction With Feedback Enhances INS

#### 4.1.1 Turn‐Taking and Bidirectionality

In our study, whether in ISC or ISFC analyses, we consistently found that interactions involving feedback tend to show higher INS compared to those without feedback. During a conversation, the neural processes supporting speech production and perception overlap in time and, based on context, expectations and the dynamics of interaction, they are also continuously modulated in real time (Mukherjee et al. 2019). The primary distinction between interactions with and without feedback lies in bidirectional verbal responses during dialogue, as opposed to unidirectional transmission of verbal signals during monologue, which is mainly influenced by turn‐taking behavior during conversation. The experimental design incorporated scenarios both with and without feedback, assigning different roles to the interacting participants. It was observed that when the participants assumed different roles, the neural interactions between participants' brains exhibited notable differences (Li et al. 2024).

#### 4.1.2 Rhythmic Coordination and Speech–Brain Coupling

Early research has shown that turn‐taking is a ubiquitous and highly coordinated phenomenon involving synchronization of endogenous oscillators in the brains of speakers and listeners based on the speaker's speech rate in conversation (Wilson and Wilson 2005). A prior VI hyperscanning study observed higher INS during a turn‐taking number counting task (Ahn et al. 2018). This suggests that even verbal responses without special semantics can lead to INS, emphasizing the importance of turn‐taking responses. Previous research has found that during verbal narration, the brain oscillations of the speaker and listener are synchronized (Hirsch et al. 2018). To some extent, INS is mediated by low‐level neural mechanisms of speech‐brain coupling and is also influenced by interactive processes that occur within the verbal context.

#### 4.1.3 Generalization and Interpretation

A hyperscanning study on joint actions showed that statistically significant correlations between different brain signals do not depend on direct physical communication between the brains. Instead, it can be seen as an indication of an indirect chain of events that starts from specific brain regions belonging to the first subject and ends in the cerebral processes elicited in brain areas of the second subject (Astolfi et al. 2014). The level of between‐brain neural coupling reflects speaker‐listener alignment at different levels of linguistic and extralinguistic representation (Schoot et al. 2016). Our findings reinforce this idea, supporting that interactions with feedback have higher INS than those without. This suggests that real‐time bidirectional information exchange that involves turn‐taking behavior, rather than transmission and reception of unidirectional information, leads to stronger INS. Our results showed that in both OVI and OTI, higher INS were found in interaction conditions with feedback. We thus conjecture that this phenomenon is universal across different interaction conditions, including CAOI and FVI. INS likely reflects similarities in brain response and shared representations driven by common stimuli. Therefore, in the context of interdependent social interaction, INS ostensibly reflects information exchange (Newman et al. 2024).

### 4.2 The Involvement of Visual Contact in VI Enhances INS

#### 4.2.1 Visual Contact and Multimodal Cues

In our study, we found that the F2F/ViC interactions showed higher INS than the B2B/VoC interactions. Fundamental distinction between F2F/ViC and B2B/VoC lies in whether visual contact evolved significantly between the two interacting individuals. Visual contact goes beyond the input of visual information; it involves the multi‐modal information fusion, including facial expressions, body language, and other forms of nonverbal interaction. Studies found that during verbal and nonverbal interactions between participants, eye contact can lead to an increase in INS (Chen et al. 2020; Kinreich et al. 2017).

#### 4.2.2 Engagement of the Social Brain Network

The brain regions involved in eye contact overlap with the structure of the social brain network, including the prefrontal cortex, the superior temporal gyrus, the fusiform gyrus, and the cingulate gyrus. This suggests that mutual gaze communication is crucial for inferring others' emotions and intentions, serving as a key factor in successful interactions. Furthermore, a study found that direct eye contact results in a higher phase synchronization between the brains of parents and infants (Leong et al. 2017). In a study on the impact of eye contact on the frequency, direction of INS, as well as the corresponding network characteristics, it was found that eye contact significantly influences INS. This indicates that eye gaze communication serves as an intrinsic social signal (Luft et al. 2022).

#### 4.2.3 Studies on Shared Gaze and INS

Existing interbrain studies have demonstrated the significant inducing effect of shared gaze on INS (Endevelt‐Shapira et al. 2021; Hirsch et al. 2017; Kinreich et al. 2017). Consistent with the biobehavioral synchronization model (Feldman 2012, 2021), INS is associated with shared gaze and empathetic engagement, but not during ViC. Hyperscanning studies have found increased connectivity between frontal and temporal lobe during F2F involving shared gaze (Hirsch et al. 2017); INS has been found to be embedded in gaze synchronization moments (Dikker et al. 2021; Kinreich et al. 2017); F2F interactions involving shared gaze triggered more INS than prefilmed videos of facial interactions (Jiang et al. 2016). Studies have shown that clips of shared gaze can enhance neural coordination by supporting the communication of social signals, predicting ongoing intentions, recognizing a partner's emotional state, and the ability to execute shared goals (Schilbach et al. 2013; Tang et al. 2016).

### 4.3 The Impact of FVI and CAOI on INS

CAOI has emerged as a topic of significant public interest, driven by the rapid advancement of social communication technology and the unprecedented changes brought about by the COVID‐19 pandemic (Schwartz et al. 2022). However, emergent evidence suggests that virtual interfacing may not be equivalent to F2F interactions (Balters et al. 2020; Lu and Pan 2023).

Our study delves into these evolving dynamics, exploring the differences between CAOI and FVI. We found that both F2F and CAOI interactions can elicit neural coupling between interacting partners. However, our results also highlight a notable distinction: CAOI tends to reduce the level of INS typically observed in natural social interactions involving physical co‐presence. This reduction suggests that while CAOI can facilitate communication and maintain social bonds, it may not fully replicate the depth and richness of in‐person interactions. This provides some insights suggesting that communication should ideally avoid remote online video or voice calls. Similarly, Balters et al. found that video conferencing led to a decrease in conversational turn‐taking behavior (Balters et al. 2023).

Furthermore, the results exhibited different activation patterns during CAOI compared to F2F interactions. In contrast, online interactions, especially those mediated by video (ViC) and text (TWE), activated distinct interbrain networks, with varying degrees of involvement in different brain areas.

These findings have important implications for understanding the impact of CAOI. As society continues to integrate digital communication tools into daily life, it is crucial to consider how these technologies shape our social experiences and cognitive processes.

### 4.4 Neural Correlates of F2F, TWE, and ViC

We compared the inter‐brain connectivity networks across the three conditions (F2F, TWE, and ViC) and conducted an in‐depth analysis of their underlying neural mechanisms. F2F communication elicited more widespread neural activation across multiple brain regions. Although ViC showed a similar pattern, it still exhibited notable differences compared to F2F. During F2F interaction, the subject not only received auditory and visual signals but also instantly captured subtle changes in facial expressions and vocal intonation (Cañigueral et al. 2021). Although ViC also conveys substantial information, some subtle bodily cues may not be fully captured in this mode. Close physical presence during F2F interaction facilitates the perception of a wider range of subtle social signals, such as body posture and movement, which are often less accessible in virtual communication (Drijvers and Holler 2022; Jiang et al. 2012). This mechanism also helps explain why TWE corresponds to weaker inter‐brain connectivity: due to the unimodal nature of text‐based information, the brain generally only needs to engage basic functional regions for processing. Overall, we propose that the hierarchy of brain activation across the three communication modes fundamentally reflects differences in information processing complexity. Richer multimodal information requires the recruitment of more neural resources for integrated processing, and the robust co‐activation of multiple brain regions between individuals serves as one key mechanism enhancing inter‐brain synchronization. Conversely, simplified information exchange elicits only limited neural activation, resulting in inter‐brain synchronization that is too weak to be reliably detected (Schwartz et al. 2024).

### 4.5 Discuss the Unexpected Results

In our study, we discovered some unexpected results. First, when calculating ISC, we found that OTI (TWE, TO) exhibited stronger ISC compared to online and FVI (Figure 3A,B). However, this pattern was not observed in brain network analysis. ISC primarily measures the similarity of neural activity between different subjects during the same time intervals (Hasson et al. 2004; Nastase et al. 2019). This method focuses on temporal synchronization and can capture transient, shared response patterns. In contrast, brain network analysis uses frequency domain methods and pays more attention to functional connectivity between different brain regions and its stability (Sänger et al. 2012). Therefore, a higher ISC does not necessarily link to stronger ISFC, since ISC reflects shared temporal responses, whereas ISFC emphasizes frequency‐resolved spatially interbrain connections and may depend on fewer but more coherent neural connections (Hu, Ruan, et al. 2025; Hu et al. 2023). In addition, VIs involve more significant facial muscle movements, and certain noise or artifacts might be amplified in one type of analysis but filtered out in another.

Second, we found that in F2F and B2B interactions without feedback, F2F showed significantly lower INS compared to B2B (Figure 3A,B), contrary to the general expectation that interactions with visual cues yield higher INS than those without. This phenomenon can be explained through the mechanism of emotional avoidance. Emotional avoidance refers to individuals' tendency to diminish their attention and processing of these emotional signals when confronted with intense emotional stimuli or social pressures, in order to avoid experiencing negative emotional reactions (Gyurak et al. 2011). During F2F interactions with feedback, participants can directly observe each other's facial expressions, eye contact, and body language, which are nonverbal emotional cues. These cues may trigger strong emotional responses and increase the engagement and emotional resonance between the participants. However, in F2F interactions without feedback, humans' strong focus on other individuals' attention and activity is provocative (Shepherd et al. 2010). To protect themselves from potential emotional distress, participants might unconsciously adopt strategies of emotional avoidance, reducing their attention and processing of these emotional cues (Hess et al. 1997; Kang and Wheatley 2015). Thus, such behavior leads to a reduction in INS. In contrast, B2B interactions reduce direct exposure to emotional cues, thereby lowering the likelihood of emotional avoidance.

Beyond the interpretation of emotional avoidance, an alternative explanation may come from cognitive load theory, particularly the concept of extraneous cognitive load. According to this theory, extraneous load arises not from the intrinsic complexity of the task itself, but from the way information is presented and the nature of participant engagement required by the task design (Sweller et al. 2019). In the F2F condition without feedback, the interaction format may have imposed an unnatural and cognitively taxing environment: the listener was confronted with rich displays but was restricted from providing any verbal or nonverbal response. Similarly, the speaker was tasked with narrating to a physically present partner who remained passive and unresponsive throughout the interaction. This asymmetrical and socially incongruent setting may have increased extraneous load for both participants, thereby consuming neural resources that would otherwise support interpersonal alignment and ultimately reducing INS.

Additionally, for OTI without eye contact, we added emojis to the plain text interactions to incorporate more visual cues, considering that text interactions using emojis are also widely popular in mobile social chat applications. However, our study did not reveal significant differences between TO and TWE conditions. This could be attributed to the fact that the TO task requires visual attention during reading and typing, and the few emojis used in the TWE condition may not have been sufficient to induce significantly higher INS compared to the TO condition. The emphasis on ecological validity in our study led to uncontrollable variables, such as the type and frequency of emoticon usage.

### 4.6 Limitation and Outlook

Despite achieving significant insights into the differences in neural synchronization between OVI and FVI forms, our study has several limitations that warrant attention: First, the sample was primarily focused on a specific age group, failing to represent a wide range of age groups. Different age groups may exhibit notable variations in cognitive processing, communication styles, and technology usage habits, which could influence INS. Second, the participants in this study were all laboratory mates with ordinary levels of intimacy, limiting our understanding of neural synchronization mechanisms under various types of intimate relationships. The degree and nature of intimate relationships, such as friendships, family ties, or romantic partnerships, can profoundly affect neural synchronization patterns during communication.

Therefore, future research should address several key areas. First, it should incorporate a more diverse age spectrum to comprehensively evaluate the impact of age on INS within different VI forms. Secondly, to delve deeper into this issue, subsequent studies should explore the specific impacts of different levels of intimacy on INS within VI contexts. In addition, although our research systematically manipulated interaction conditions such as feedback presence and visual access, we did not directly include behavioral measurements such as speaking time, turn‐taking frequency, eye gaze tracking, emoji usage patterns, and textual metrics (e.g., message length). These indices could serve as important correlates to neural synchronization; thus, future work will consider integrating such data to better understand neural patterns. Lastly, future research should also investigate strategies to improve CAOI quality, potentially bridging the gap between online and offline interactions to foster stronger and more satisfying social connections.

## 5 Conclusion

This study elucidates the complex interplay among VIs, feedback, visual contact, and neural synchronization. Comprehensive experiments encompassing both verbal and nonverbal interactions were designed, which validated the significant differences between online and offline communications. Leveraging these findings, we extended the role of feedback and visual information—specifically in enhancing brain‐to‐brain synchronization—from FVIs to CAOIs. These insights offer valuable understanding into the neural correlates underlying both natural and technology‐mediated communication.

## Author Contributions

Piqiang Zhang: software, data curation, investigation, validation, formal analysis, visualization, writing – original draft. Yuhao Fang: software, data curation, investigation, validation, formal analysis, visualization, writing – revision. Xiao Gong, Lihao Fu, Enguang Ma, Debin Zhou: software, data curation. Shiang Hu, Zhao Lv, Pedro A. Valdes‐Sosa: conceptualization, formal analysis, visualization, supervision, project administration, writing – review and editing.

## Funding

This work was supported by the National Natural Science Foundation of China (62101003), Natural Science Foundation of Anhui Province (2508085MF156), and Chinese Ministry of Education (MOE) Project of Humanities and Social Sciences (22YJC190007).

## Data Availability

The data that support the findings of this study are available on request from the corresponding author. The data are not publicly available due to privacy or ethical restrictions.
